# Characterization of the Regenerative Capacity of Membranes in the Presence of Fouling by Microalgae Using Detergents

**DOI:** 10.3390/membranes16010007

**Published:** 2025-12-26

**Authors:** Volker Bächle, Marco Gleiß, Hermann Nirschl

**Affiliations:** Institute of Mechanical Process Engineering and Mechanics (MVM), Karlsruhe Institute of Technology (KIT), Strasse am Forum 8, 76131 Karlsruhe, Germany

**Keywords:** membranes, microalgae, fouling, regeneration, backwashing, detergents, enzymes

## Abstract

The filtration of microalgae generates fouling through algal matter and exopolymer particles with consequences for the flow rate. Therefore, regeneration that is as complete and continuous as possible is necessary. For this purpose, a commercial membrane with a pore size of 0.8 µm is contaminated with the microalgae mixture Haematoccocus Pluvialis and Tetradesmus obliquus, and then regenerated with mechanical (backwashing), chemical (HCl, NaOH, NaClO, P3-Ultrasil) and biological (dishwashing and laundry detergents) cleaning methods. The filtration time of the individual experiments is compared with a new membrane, and the increase is determined. Backwashing cleans the pores, but the biofilm sticks to the membrane surface and blocks the pores shortly after a new cycle. It was shown that the biofilm can only be removed chemically through oxidative effects or anionic surfactants. Hydrolysis does not remove the biofilm, and it can actually make the blockage worse. Bigger cellular residues can only be removed with enzymes. This improves cleaning performance by 61% compared to commercial cleaning agents for membranes and 42% compared to backwashing.

## 1. Introduction

Microalgae, as phototrophic microorganisms and versatile raw materials, have become increasingly important in recent years. This is partly due to the fact that around 50% of carbon dioxide fixing and 50% of oxygen production is carried out by microalgae [1,2,3,4]. An approximate number of 200,000 to several million species is estimated, but only between 40,000 and 60,000 species have been recorded to the present day [5]. One example of this is the microalgae *Schizochytrium* sp. that can be cultivated for omega-3 docosahexaenoic acid production. However, the color pigments, antioxidants and polyunsaturated fatty acids used in the food and animal feed industry are also important, as these sale markets represent great economic potential [6,7,8]. Low demands for water quality facilitate extraction in arid regions by means of water recirculation [9].

There are two limiting steps in the production of microalgae. On one hand, the aim is to achieve the highest possible biomass concentration during cultivation and, on the other hand, to carry out efficient and energy-saving solid–liquid separation. This paper deals with realizing the most efficient downstreaming possible. During the continuous filtration process, the filter media resistance increases and the permeability decreases as the number of cycles increases. These phenomena are caused by various fouling mechanisms of the microalgae; as a result of these, the active filter surface decreases and the filtrate flow decreases [10]. The replacement of these membranes is cost-intensive, while the fabrication involves the use of several fossil-fuel-derived chemicals, which results in both resource depletion and greenhouse gas emissions [11,12]. Continuous membrane regeneration is therefore necessary to prevent fouling or to regenerate fouling that has occurred [13,14,15].

The solid concentrations that can be achieved in the cultivation of microalgae suspensions are generally below 2 g L^−1^ [16]. The separation task proves to be complex, as the particles are present in the μm range and with a small difference in density to the liquid phase. As an example, centrifuges with high Z-values can be helpful. The disadvantage of this is that the energy required to accelerate the fluid represents 20% to 30% of the overall process [17]. It has been estimated that 90% of the total cost of algae biomass production may be attributed to harvesting and dewatering [18], with dewatering contributing between 60% and 70% of the total cost [19,20,21,22]. A more energy-efficient alternative for separation is filtration using driving potential. Additional energy savings when using a vacuum pump can be achieved by using gas-impermeable hydrophilic membranes [23]. The challenge with membranes is regeneration. Chemical, physical and biological cleaning methods are used.

Fouling can be removed from the membrane surface by using physical cleaning based on electrical field, hydraulic and mechanical forces. The electrical field method creates in situ antifouling conditions by charging particles electrically and applying field forces opposite to the flux direction [24]. One mechanical approach to minimize fouling is the usage of novel spacers as a structural component to minimize the pressure drop [25,26]. Another mechanical approach is the backwashing method, in which the membrane is flushed from the opposite direction and the fouling substances are removed by shear forces [27]. Chemical cleaning agents are also used to support the detachment behavior of the fouling substances. Chemical cleaning is achieved by overcoming the adhesive forces or by dissolving the fouling substance [27]. For example, Zhou et al. were able to reduce the fouling rate by 50% by adding 0.01 mol L^−1^ NaOH during backwashing, compared to backwashing with pure water [28]. Maskooki et al. compared chemical cleaning with physical cleaning, using ethylenediaminetetraacetic acid and simultaneous ultrasonic treatment [29]. They were able to regenerate the hydraulic resistance from 14.2% after fouling to 66.9% after treatment, compared to the new membrane. Pure chemical cleaning only achieved a regeneration of 27.4% and ultrasonic cleaning achieved 41.9%. If sensitive membranes are used that are neither chemically nor mechanically robust, biological cleaning is relevant [13]. Here, enzymes are primarily used, which metabolize biofouling components in a highly specific manner on the basis of substrate catalysis [30]. Munoz-Aguado et al. investigated the purification efficiency with and without Terg-A enzymes in membranes contaminated with wheat proteins. It was possible to restore 91% of the original flux of the membrane [31]. Chen and Columbia used alginate lyase to regenerate alginate fouling. They were able to reduce the fouling resistance by up to 85% [32]. One disadvantage, however, is the long exposure time of the enzymes for the decomposition of the foulant. Furthermore, there are few studies on the regeneration of complex fouling, based on a combination of oils, proteins and cell residues. The aim of the study is therefore to gain a more precise understanding of the regenerative capacity of membranes in the fouling of biological microparticles and the capability to establish biological cleaning in a continuous process. Above all, the overlapping of a roller discharge with cake discharge can influence regeneration, as roller forces can press small biological components into the pores, thereby preventing contact with the cleaning agent. In addition, a direct comparison of different cleaning methods (mechanical, chemical, and biological) should be carried out in order to test the feasibility of filtration under industrial conditions. When dealing with microalgae, the cleaning potential is to be optimized in order to ensure the most efficient filtration possible.

Part of this study is the observations of mechanical, chemical and chemical–biological regeneration. Due to the individuality of biological microparticles, the effects of different cleaning agents are different for each product. A cleaning strategy that removes the majority of fouling caused by microalgae is therefore of particular interest [13,33]. The chemical cleaning agents hydrochloric acid, sulphamic acid, sodium hydroxide, sodium hypochlorite and an alkaline industrial cleaner are tested in concentrations according to the manufacturer’s instructions and values from the literature, with slight variations. As chemical–biological cleaning agents, powdered dishwashing and laundry detergents are tested with slight variations in temperature. For identical framework conditions, all test series are carried out with new membranes and pore sizes of 0.8 µm. The quantification of regeneration is based on the increase in filtration time in relation to the first filtration of a new membrane.

## 2. Materials and Methods

### 2.1. Materials and Sample Preparation

This study investigates cake filtration using a mixture of the microalgae *Haematococcus pluvialis* and *Tetradesmus obliquus* from Sea & Sun Organic GmbH (Trappenkamp, Germany). The microalgae mixture has an average particle diameter of x_50.3_ = 10.13 μm with x_10.3_ = 2.26 μm and x_90.3_ = 30.42 μm as the lower and upper particle sizes, determined by laser diffraction spectroscopy on an LBS Helos Quixel from Sympatec GmbH (Basel, Switzerland). According to Lorenz, the genus Haematococcus contains an average content of 23.6% protein, 38% carbohydrates and 13.8% lipids [34]. In the Tetradesmus genus, do Carmo Cesário et al. identified 61.6% unsaturated fatty acids, 3.4% saturated fatty acids and 28.5% protein in the dry biomass [35]. The remaining components of both microalgae are calcium, magnesium, various vitamins, carotene, chlorophyll and amino acids. To accelerate biofilm formation on the membrane, the suspension is frozen and thawed twice. The resulting cell lysis produces free algal organic matter (AOM) and transparent exopolymer particles (TEP) in the suspension [10]. In addition, a mixture of two different green algae leads to a more complex fouling problem, as the smaller cells cause even more fouling mechanisms (coating formation, internal adsorption, pore blockage, biofouling) to overlap [36]. The cleaning strategies developed thus counteract not only fouling from monocultures, but also other contaminants that occur. In all experiments, a bio-dry mass concentration of 1 g L^−1^ is used, which is determined gravimetrically by drying. The temperature during the experiments is kept constant at 20 °C, except for biological–chemical cleaning. In these cases, the temperature varies between 20 °C and 60 °C.

A commercial TrakEtch^®^ (PET/PET) 0.8 nuclear track membrane from SABEU GmbH & Co. KG (Northeim, Germany), with a pore diameter of 0.83 ± 0.01 µm, is used for filtration. The pore diameter is determined by using a Capillary Flow Porometer CFP–1500–AEX from PMI (Ithaca, NY, USA). For this purpose, samples with a diameter of 22 mm are used with silicone oil AK 10TM from Wacker Chemie AG (Munich, Germany) with a surface tension of *γ* = 20 mN m^−1^ and the contact angle is assumed to be Θ = 0°, due to complete wetting. The equation to determine the pore size is defined in Equation (1) with *P_cap_* as the capillary pressure [14].

(1) $$d_{\mathrm{cap}}=\frac{4\cdot \gamma_{L}\cdot \cos\theta }{P_{\mathrm{cap}}}$$

The hydrophilic nuclear track membrane has a pore density of 50 ± 7 10^6^ cm^−2^, an air throughput of 17 ± 4 min^−1^ cm^−2^ bar^−1^ and a membrane thickness of 150 ± 50 µm. The membrane is equipped with a support fabric for greater mechanical stability. The membrane and support fabric are made of polyethylene terephthalate (PET). To ensure a flat surface, the membrane is placed on another support fabric, TETEX^®^ MONO 07-1100-SK 020 from Sefar AG (Bülach, Switzerland), which is also made of PET.

The membrane is cleaned mechanically, chemically, and biologically–chemically. During mechanical cleaning, deionized water is flushed in the opposite direction of filtration at pressures between 0.2 and 0.8 bar (backwashing). Chemical cleaning involves the use of hydrochloric acid (HCl) and sulfamic acid (H_3_NSO_3_) as acidic cleaning agents at a concentration of 0.5 mol L^−1^. Simple acids have a hydrolytic effect on inorganic compounds and are therefore considered to be one-dimensional cleaning agents. For one-dimensional cleaning in the alkaline range, sodium hydroxide (NaOH) is also used at a concentration of 0.5 mol L^−1^ for comparability. In the alkaline range, organic compounds undergo hydrolysis. In addition to cleaning by hydrolysis, the oxidation of biological substances is investigated by using sodium hypochlorite (NaClO) with concentrations in the range of 0.5–2 mol L^−1^. This concentration range was chosen because the experiments are then comparable with the results of Ding et al. [37]. All acids and alkaline solutions are from Carl Roth GmbH + Co. KG (Karlsruhe, Germany). By comparing all simple acids and alkaline solutions, the main cause of microalgae pollution can be determined.

In addition to one-dimensional cleaning agents, P3-Ultrasil 112 from Ecolab Deutschland GmbH (Monheim am Rhein, Germany) is used as a multidimensional cleaning agent in concentrations ranging from 0.1 to 1% *w*/*w*. Multidimensional cleaning agents consist of several components, which means they combine different applications, such as hydrolysis, oxidation, emulsification and complex formation. P3-Ultrasil is an industrial cleaning agent for membranes for the removal of biological residues. Its main component is 5–10% *w*/*w* NaOH, which means it belongs to the group of alkaline cleaners. Other components include 2.5–3% *w*/*w* sodium dodecylbenzene sulfonate as an anionic surfactant and 2.3–3% *w*/*w* sodium cumenesulfonate to increase the solubility of surfactants. For biological–chemical cleaning, a dishwashing detergent and a laundry detergent from Chemolux Germany GmbH (Düsseldorf, Germany) are used, each with a concentration of 5 g L^−1^. This corresponds to the manufacturer’s instructions for cleaning dishes. In addition to ingredients for hydrolysis, oxidizing agents, surfactants and defoamers, the two powdered cleaners also contain enzymes for breaking down and dissolving protein-based residues, oils, lipids and fats [38]. Since enzymes are temperature-dependent, a temperature variation between 20 and 60 °C is carried out at a constant concentration of the cleaning agent and an exposure time of 5 s [36].

### 2.2. Experimental Setup and Procedure

**Figure 1:**
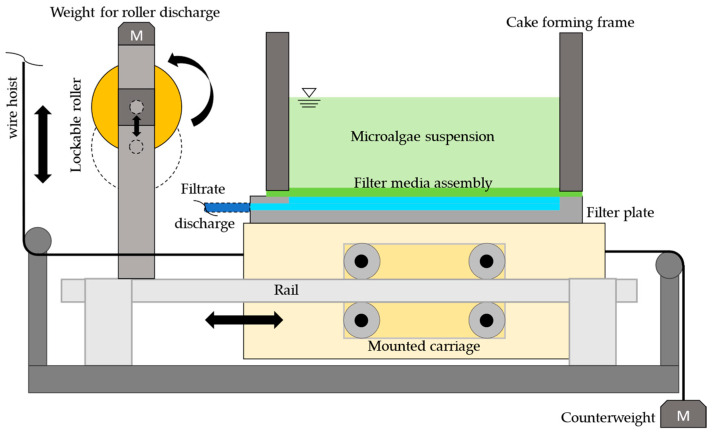
Side view of the experimental setup.

The results of this paper serve to characterize continuous cleaning on a vacuum drum filter, based on a previous publication [39]. In a vacuum drum filter, the membrane is attached to the outer surface of the drum, and the drum is partially immersed in the suspension with its outer surface. Thus, the filter cake is formed when immersed in the suspension and dewatered when the filter cake emerges, and then the membrane is cleaned before being immersed again. Accordingly, the exposure time of the cleaning agents is limited. The exposure time is therefore defined as a constant 5 s for all experiments. The existing experimental setup (Figure 1) simulates filtration in a vacuum drum filter. The filter plate has an active filter area of 72 cm^2^, through which the filtrate is fed into a water separator by means of a vacuum pump at a pressure difference of 0.8 bar. The water separator is weighed continuously to determine the filtrate flow. The cake formation frame is pressed onto the filter plate using screw connections and sealed at the bottom. The microalgae suspension can be poured into the cake formation frame and filtered through the membrane. For cake removal using a roller, according to Bächle et al. [39], the structure is mounted as a mounted carriage on a rail. This moves via a drag device with a corresponding counterweight. The rubberized roller has a diameter of 90 mm and a Shore A hardness of 35°. The line pressure can be controlled via the mass attached to the structure and is 215 N m^−1^ for roller discharge. The line pressure force is defined as the linear pressure with which the roller presses on the membrane and is defined according to Equation (2). Here, $m_{B}\;$ describes the mass of g, the gravity with 9.81 m s^−2^, and *L*, the roller length [40].

(2) $$q_{p}=\frac{m_{B}\cdot g}{L}$$

**Figure 2:**
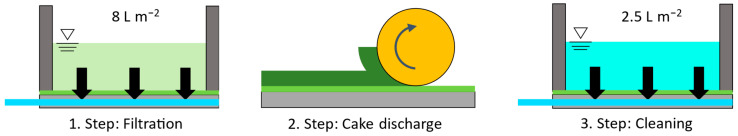
Schematic illustration of a cycle comprising the three steps of filtration, cake discharge using a roller and membrane cleaning. In filtration, a constant volume per filter area of suspension $V_{\mathrm{sus}}$ of 8 L m^−2^ is used, and a constant volume of 2.5 L m^−2^ is used for cleaning.

The test procedure can be divided into three steps, see Figure 2. The first step involves the filtration of 8 L m^−2^ and the cake formation on the cake-forming frame. The quantity of 8 L m^−2^ corresponds to the filtration quantity of one cycle, based on previous work on the laboratory vacuum drum filter [39]. To do this, the microalgae suspension is placed on the membrane and, once a vacuum of 0.8 bar has been reached, a valve on the filtrate side is opened. The filtrate flow is then measured gravimetrically. Once this flow stops increasing, filtration is complete. The filtration time is defined as the time required to filter between 10% and 90% weight of the final value of the filtrate. Comparisons of the filtrations are made using the normalized filtration time, $t^{*}$, to the respective cycle, $t_{\mathrm{cycle}}$. This is defined by Equation (3).

(3) $$t^{*}=\frac{t_{\mathrm{cycle}}}{t_{0}}$$

Here, $t_{\mathrm{cycle}}$ is the filtration time for an individual cycle and $t_{0}$ is the filtration time for the first cycle with a new membrane. In addition to the filtration time, the comparison can also be represented using the normalized flux for each cycle, ${\dot{V}}_{\mathrm{cycle}}$. In this case, a constant volume, $V_{\mathrm{sus}}$, is filtered and then related back to the first cycle, ${\dot{V}}_{0}$, in order to calculate the normalized flux, ${\dot{V}}^{*}$, according to Equations (4) and (5).

(4) $${\dot{V}}_{\mathrm{cycle}}=\frac{V_{\mathrm{sus}}}{t_{\mathrm{cycle}}};\;V_{\mathrm{sus}}=\mathrm{const}.=8\;{L\;m}^{-1}$$

(5) $${\dot{V}}^{*}=\frac{{\dot{V}}_{\mathrm{cycle}}}{{\dot{V}}_{0}}$$

After filtration of the microalgae suspension, the filter cake is removed in a second step. To do this, the cake formation frame is removed and the roller is placed on the membrane with a line pressure of 215 N m^−1^. The roller rotates at the same circumferential speed of 200 mm min^−1^ as the mounted carriage moves. With these settings, a shear force of 11.36 N results in the opposite direction discharge, according to Bächle et al. [39]. The shear force is determined from the difference in power consumption between no-load and contact stress of the motor (MFA–Como drills, 919D Series). With the efficiency of the motor and the rotation speed of the roller, the torque, and thus the shear force on the membrane, can be calculated.

For the subsequent cleaning in the third step, the cake-forming frame is reattached. A constant quantity of 2.5 L m^−2^ of cleaning agent is applied to the membrane. This quantity is required to completely wet the membrane after the roller has been removed by the cleaning agent. After a 5 s exposure time, the vacuum is applied again and the cleaning agent is drawn through the membrane. The next filtration cycle begins immediately after successful filtration of the cleaner by applying another 8 L m^−2^ of the suspension to the membrane. A new membrane is used for each test series.

## 3. Results

### 3.1. Filtration Without Cleaning Agents

**Figure 3:**
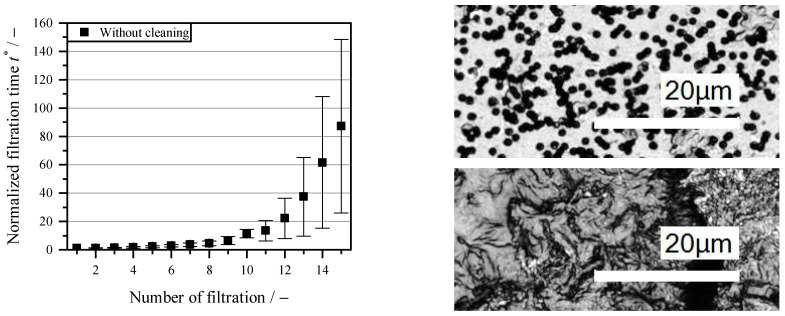
Increase in filtration time $t^{*}$ relative to cycle 1 (Equation (3)), during filtration of the microalgae suspension without cleaning (**left**) and comparison of a new membrane (**top right**) and after coating formation by algae residues after 15 cycles (**bottom right**) under the microscope.

To illustrate the problem of fouling, the left diagram in Figure 3 shows the increase in filtration time without cleaning as a function of the number of filtration cycles. With an applied pressure difference in Δp = 0.8 bar, the solid phase of the microalgae suspension (1 g L^−1^) is separated from the liquid phase. The filtration time at a constant suspension volume of 8 L m^−2^ increases exponentially with the increasing cycle numbers and increases 87-fold from the 1st cycle to the 15th cycle. The increase in filtration time with each cycle is particularly noticeable. Various fouling mechanisms occur, including surface layer formation, pore blockage and internal absorption. The surface layer can be partially removed by the roller, which applies shear forces from the roller surface to the membrane surface. Therefore, only pore blockage and internal absorption are responsible for the decrease in flow. However, for this to happen, the roller surface must be perfectly parallel to the membrane over its entire length, which is not possible due to manufacturing tolerances. This results in significant variations in surface layer formation after a greater number of cycles. This biofouling consists of algal organic matter (AOM), algal cells and transparent exopolymer particles (TEP) in the mixed suspension [41]. The right side of Figure 3 shows microscope images of the examined membrane. All pores are visible on the new membrane, whereas no pores are visible on the used and uncleaned membrane. The active filter area of the membrane is disrupted by a gel layer that has formed. This leads to an increase in the filter medium resistance, which reduces the filtrate flow and requires the membrane to be replaced to ensure the continuity of the process. Thus, due to the broad particle size spectrum and the vitality of the organic particles, a complex fouling problem arises [10]. The resulting uneconomical nature of the process can already be seen after 10 cycles, with an 11-fold increase in filtration time. The scope of subsequent tests is therefore 10 cycles, as fouling already occurs during this period with the suspension used and must be removed.

### 3.2. Mechanical Regeneration Through Backwashing

**Figure 4:**
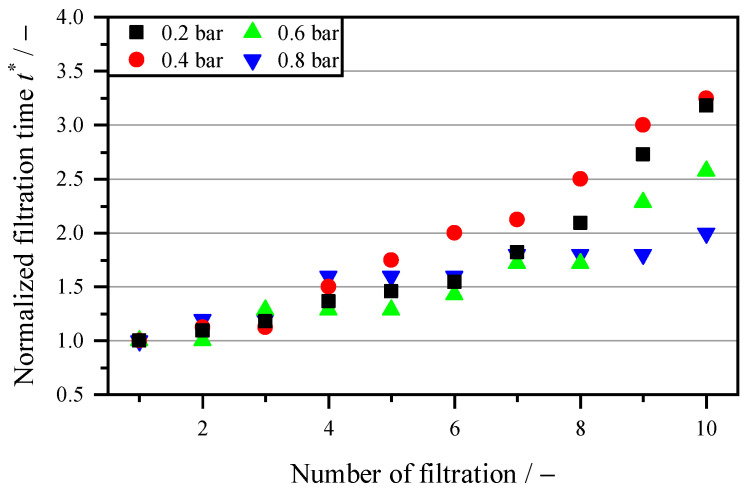
Increase in filtration time $t^{*}$ (Equation (3)) with backwash volumes of 8 L m^−2^ water, with backwash pressures between 0.2 bar and 0.8 bar for each filtration cycle.

The aim of regeneration is to clean the membrane in order to keep the filtration time and thus the throughput constant for subsequent filtration cycles. For comparison purposes, the various cleaning methods are compared mechanically with backwashing, chemically, and biologically. Figure 4 shows regeneration with backwashing. The backwash fluid is tap water with a backwash volume of 8 L m^−2^, which is passed through the membrane at pressures between 0.2 and 0.8 bar. The backwash volume thus corresponds to the filtration volume, which makes the process uneconomical. Mechanical regeneration is only used here as a comparison with chemical and biological–chemical cleaners, which means that economic efficiency is not taken into account. In contrast to chemical and biological regeneration, a support structure is attached to the fabric for the mechanical stabilization of the membrane. Compared to Figure 3, which shows filtration without cleaning, after 10 cycles with a factor of 11.37, there is an increase in filtration time at 0.2 bar by a factor of 3.18. This means that the filtration performance has increased by 357%. This value increases further with higher backwash pressures. However, when the backwash pressure is increased fourfold, the filtration time after 10 cycles decreases from 318% to 180%. The increase in filtration time follows an exponential curve, which is due to an increase in irreversible fouling. Fats, oils and lipids, in particular, form a gel-like layer that can be removed from the pores by backwashing, but the layer remains on the surface of the membrane.

Compared to the literature, Psoch and Schiewer [42] achieved a doubling of the flow rate at a backwash pressure of 0.45 bar, which would correspond to a reduction of 50% in the filtration time. In this study, the flow rate between backwashing and without cleaning at 0.4 bar is 350% at the end. However, Psoch and Schiewer [42] used only 3% of the backwash volumes used in this study.

### 3.3. Influence of Different Chemical Cleaning Agents

**Figure 5:**
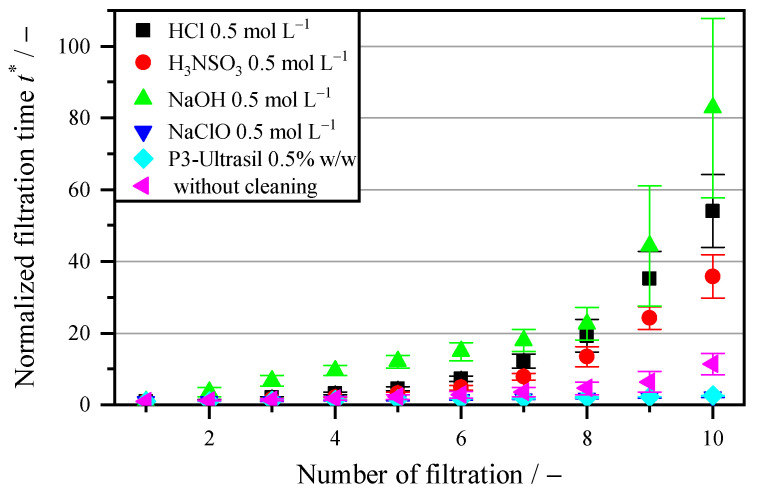
Increase in filtration time $t^{*}$ (Equation (3)) with regeneration, using different chemical cleaning agents and an exposure time of 5 s after each cycle.

When attempting chemical regeneration of the membrane, simple acids are used first. Figure 5 shows the curves for the various acids with a molar concentration of 0.5 mol L^−1^, which are also used in the literature [10]. These are three acids (HCl, H_3_NSO_3_, and NaClO) and two alkaline agents (NaOH and P3-Ultrasil). When comparing the acids after 10 cycles, only NaClO can be seen to have a cleaning effect. The other two acids, HCl and H_3_NSO_3_, actually increase the filtration time even further. Acids and alkaline substances generally only have a dissolving or hydrolyzing effect [36]. Inorganic compounds dissolve, particularly in acidic environments, which is beneficial for regeneration after the filtration of minerals. Organic compounds, on the other hand, do not dissolve in acidic environments. On the contrary, the acid treatment causes the existing proteins to denature. The clogging or blocking of the pores leads to an increase in filter medium resistance. Thus, when HCl is used, the normalized filtration time increase is a factor of 54.03, which is a 5-fold increase compared to the tests without a cleaning agent. Sulfamic acid, which is also used as a cleaning agent, is a strong acid with a pKa value of 1, but is weaker than HCl, which has a pKa value of −6 [43]. Accordingly, the hydrolysis effect is less, which is reflected in the lower increase in filtration resistance. Nevertheless, the increase in filtration time after 10 cycles is higher at 35.82 compared to 11.37 without a cleaning agent. No cleaning effect can therefore be achieved in the acidic range. At pH > 7, easily soluble organic compounds such as polysaccharides, oils and fats dissolve. However, at a concentration of 0.5 mol L^−1^, the membrane shows no cleaning effect. With 82.77, the increase in filtration is higher than with hydrochloric acid. In addition to the solubility of organic substances, alkaline cleaning agents also have a hydrolysis effect, which can denature proteins and cell residues in particular. This produces the same effect as hydrochloric acid. In Figure S1, the concentration of HCl and NaOH is varied slightly between 0.25 mol L^−1^ and 1 mol L^−1^. The results demonstrate that a higher concentration does not increase the flux after regeneration, due to a higher hydrolysis effect. On the contrary, a higher chemical concentration worsens the blockage of the filtration. The main cause of fouling is therefore not simple fats, saccharides or mineral components, but rather poorly soluble oils and fats that represent the biofilm, as well as proteins and cell residues.

Another important group in membrane cleaning is oxidative cleaning agents, such as sodium hypochlorite (NaClO). Oxidation causes functional groups to form in the fouling substances, which increases their hydrophilic properties. In addition, NaClO has a disinfecting effect due to the chlorine compound and counteracts biofilm formation. This can be seen in Figure 5, based on the increase in filtration time. This is 2.32 after 10 cycles, which is a factor of five less than without regeneration. This also corresponds to the cleaning effect of backwashing at 0.6 bar. However, NaClO is acidic in nature, which means that it only increases the solubility of minerals and not of poorly soluble oils or fats from algae. To compare this, tests were also carried out with an alkaline cleaning agent, P3-Ultrasil. In addition to sodium hydroxide as its main component, it also contains sodium cumenesulphonate and sodium dodecylbenzene sulfonate. Sodium cumenesulphonate helps to dissolve poorly soluble components in water and serves as a means of reducing viscosity. The addition of sodium dodecylbenzene sulfonate is an anionic surfactant and also serves to dissolve lipids from the membrane. Since P3-Ultrasil is a multi-component mixture, this is specified in mass fractions. Similarly to NaClO, it can prevent gel formation due to fouling, as fats and oils are the main causes of biofilm and these are dissolved in water by additional surfactants. However, larger proteins and cell residues are not decomposed as well here. Similar behavior is again shown in Figure 5. Here, the increase in filtration time after 10 cycles is 2.54, which roughly corresponds to the value for cleaning with NaClO. This confirms the claim that biofilm from fats and oils is the main cause of blockages.

**Figure 6:**
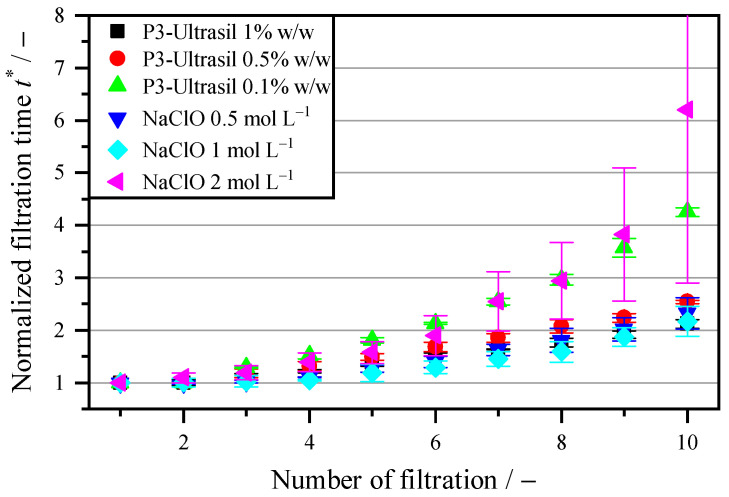
Variation in the concentration of P3-Ultrasil (alkaline) and NaClO (acidic) for characterizing the cleaning properties.

After removing the biofilm with a 0.5 mol L^−1^ NaClO and 0.5% *w*/*w* P3-Ultrasil solution, the question arises as to whether the cleaning process is concentration-dependent. Thus, the concentration was varied to characterize its influence on the membrane and biofilm removal. Figure 6 shows the increase in the filtration time of the microalgae suspension as the concentration of the cleaning agents increases. Comparing the P3-Ultrasil concentrations reveals that 0.1% *w*/*w* is insufficient for cleaning the biofilm in 5 s and regenerating the membrane. After ten cycles, the increase is 4.25 times greater, whereas the normalized filtration time is 2.54 at 0.5% and 2.12 at 1%. Doubling the concentration from 0.5% to 1.0% does not significantly reduce the filtration time. A longer exposure time could improve regenerability. However, in a continuous vacuum drum filter, this cannot be adjusted independently during filtration time, cake washing and cake discharge. For this reason, it was not investigated further. With NaClO, the filtration time decreases from a factor of 2.32 to 2.17 when the molar quantity doubles to 1 mol L^−1^. Thus, the oxidation effect is higher, and regeneration is more effective at a constant exposure time. Increasing the molar concentration of NaClO to 2 mol L^−1^ reduces the flow rate. The filtration time increases to a factor of 6.2. One possible explanation is membrane damage due to the higher chlorine content. Another possible explanation is protein denaturation due to the falling pH value, which has the same effect as with hydrochloric acid and sulfamic acid.

This is lower than the results obtained by Ding et al. He achieved 100% regeneration, using pure NaClO and additional peroxides. However, he used exposure times of 30 min and 1 h, which are not possible for continuous operation on a vacuum filter. This technology is necessary for a more complete regeneration in batch operation [44].

To characterize the blocking behavior, the membranes were dried and then the pore size distribution was determined using a porometer. The data in Table 1 shows the mass concentration of P3 Ultrasil and its influence on the average pore diameter. The higher the molar concentration of the cleaning agent, the larger the average pore diameter. However, the pore diameter still decreases slightly compared to the unused membrane with 0.83 µm. This is consistent with the results shown in Figure 6. However, there should be no significant difference between 0.5% *w*/*w* and 1% *w*/*w*. A measurement with the porometer shows a 5% reduction in the average pore size. With a further reduction in pore size to 0.66 µm at 0.1% *w*/*w*, the filtration time doubles. Moreover, the P3-Ultrasil cleaning agent has a positive effect on membrane regeneration. No trend in the average pore diameter can be observed in Table 1 for the mass concentrations of NaClO. All three mass concentrations of the cleaning agent tested showed an average pore diameter within the measurement accuracy. This contrasts with the change in flow behavior shown in Figure 6. The reduction when using 2 mol L^−1^ cannot be detected based on the average pore diameter. One possibility is that although the pores are reduced in size due to blockage, the high concentration of chlorine-containing cleaning agent causes damage to the membrane with larger pores. This can be observed below when looking at the overall pore size distribution.

**Figure 7:**
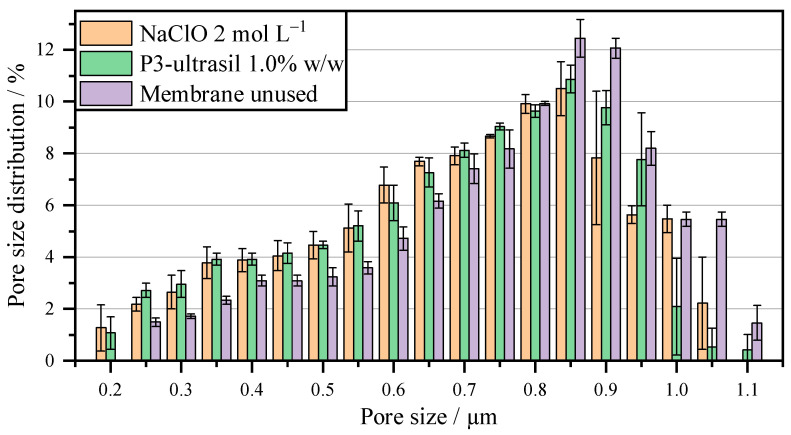
Pore size distribution of the membrane after 10 cycles with chemical cleaning after each cycle.

Figure 7 shows the pore size distribution of a new membrane, as well as the distribution after 10 cycles with cleaning between each cycle, using NaClO and P3-Ultrasil. A shift of 0.05 µm in both pore sizes can be seen. A new membrane has 1.5% of pores in the size range of 1.1 µm and 5.5% of pores in the range between 1.0 µm and 1.05 µm. The smallest pore size measured is 0.25 µm. In contrast, the smallest pore measured after regeneration with P3-Ultrasil and NaClO is 0.2 µm. The largest pore measured after cleaning with P3-Ultrasil is still 1.1 µm, but only accounts for 0.4%. With NaClO, the largest pore has a diameter of 1.05 µm. In both cases, no damage to the membrane by the cleaning agent can be detected, as the large pores in particular have not increased in diameter. Thus, all concentrations used are harmless to the PET membrane. With regard to the increase in filtration time at a substance concentration of 2 mol L^−1^ NaClO, damage to the membrane can therefore be eliminated. Since the pores have not shrunk either, there are components of the cells that shrink when the membrane dries, thus releasing the pore blockage.

### 3.4. Regeneration with Biological–Chemical Cleaning Agents

**Figure 8:**
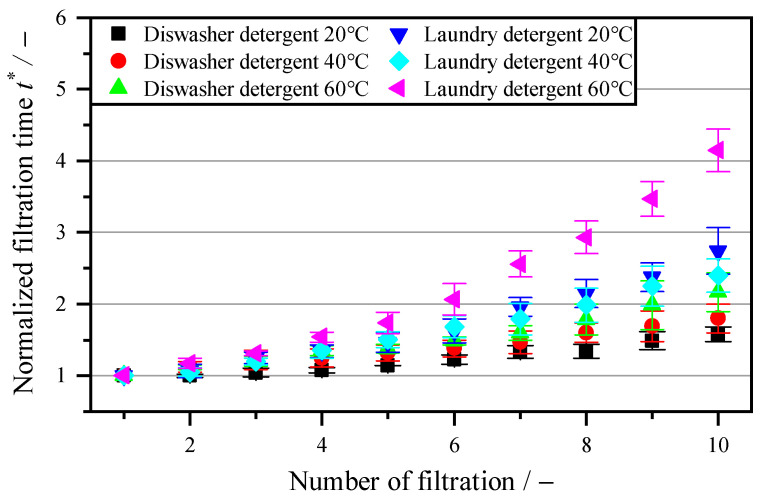
Increase in filtration time $t^{*}$ (Equation (3)) after treatment with biological–chemical cleaning agents with a respective mass concentration of 5 g L^−1^ (0.5% *w*/*w*) at 5 s exposure time.

After regeneration with purely chemical agents showed that larger cell components are difficult or impossible to dissolve, cleaning agents with enzymatic additives are now being investigated. Figure 8 shows cleaning with powdered dishwasher detergents and laundry detergents. In addition to components with an oxidizing effect from NaClO and anionic surfactants from P3-Ultrasil, these two cleaners also contain enzymes for breaking down and dissolving protein-based residues, oils, lipids and fats [38]. The solubility of fat and enzyme activity are temperature-dependent and were investigated at common temperatures in the range of 20–60 °C. The exposure time remains constant at 5 s. It can be seen that for both cleaning agents, a higher temperature results in a decrease in the flow rate, as the filtration time of 8 L m^−2^ increases. The increase in filtration time for the laundry detergent at 60 °C is 4.15, while at 40 °C, it is 240% higher than for a new membrane. A further reduction in temperature reduces the flow rate to a factor of 2.74. With dishwashing detergent, a clear trend towards lower temperatures can be seen. At a temperature of 60 °C, the filtration increase is a factor of 2.17, falling to 1.80 at 40 °C and 1.58 at 20 °C. A higher temperature then results in a lower flow rate after regeneration for both cleaning agents. This is contrary to expectations, as higher temperatures increase the solubility of oils, fats and lipids through surfactants and should therefore increase the flow rate when the exposure time on a vacuum drum filter is limited. However, higher temperatures also increase the tendency of proteins to denature, causing them to clump together and thus degrade more slowly, due to the resulting decrease in contact surface area. A longer exposure time could counteract this, but this is not possible on a vacuum drum filter without adjusting the process conditions. Therefore, this will not be investigated further.

When comparing the cleaning performance of a pure enzyme mixture, such as that described by Rudolph et al., it is helpful to use a multi-component mixture. Rudolph et al. found that the enzymes themselves can act as foulants, reducing permeability compared to normal rinsing with water. They therefore also recommended a two-step cleaning process. However, the enzyme-based cleaning agents used here do not require a two-stage process. One wash cycle is sufficient, due to the additional surfactants that prevent enzymes from being adsorbed onto the membrane surface [45].

**Figure 9:**
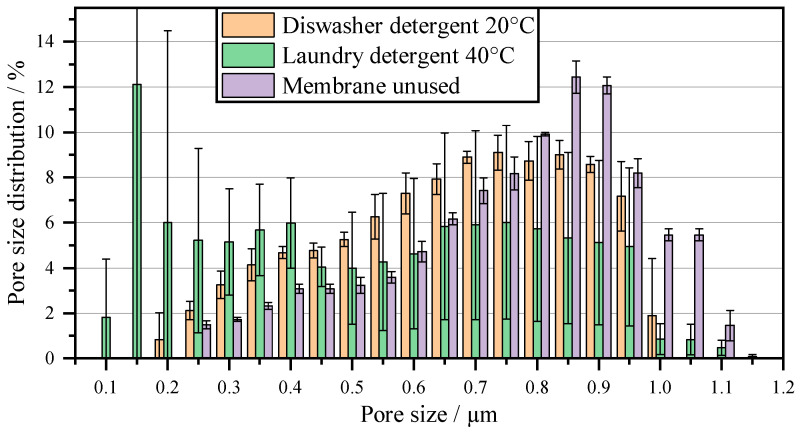
Pore size distribution of the membrane after 10 cycles with biological–chemical cleaning after each cycle.

When comparing the individual cleaning methods, biological–chemical cleaning displays the smallest increase in filtration time of 8 L m^−2^. This means that the pore size should be similar to that of a new membrane. Figure 9 shows the pore size distribution of the membrane after using dishwashing detergent and laundry detergent, which display the smallest increase in filtration time. The average pore diameter decreases from 0.83 µm for a new membrane to 0.67 µm after 10 cycles. With laundry detergent, the MFP decreases to 0.50 µm after 10 cycles of cleaning. The decrease in pore size can be explained by insoluble abrasive additives in powdered industrial cleaners, which are used to remove insoluble components, e.g., baked-on residue in dishwashers [38]. These abrasive particles are deposited in the pores and clog them. The proportion of pores < 0.4 µm has increased, especially in laundry detergent. For example, the proportion of pores with a size of 0.15 µm after cleaning with laundry detergent is 12%, and the proportion is 0% for dishwashing detergent and new membranes.

This is lower than with purely chemical cleaning, yet the flow rate is higher. This can be explained by the cell residues (cellulose, hemicellulose, pectins) on the membrane. The degree of esterification is particularly important for the solubility of pectins. Highly esterified pectin (high-methoxy pectins) requires an acidic environment for solubility, and low-esterified pectin (low-methoxy pectins) requires a neutral to slightly acidic environment. Hemicellulose, on the other hand, is insoluble in an acidic environment but can be broken down in an alkaline environment [46]. Since only one pH value can be adjusted at a time, chemical cleaners cannot remove these components. Chemical cleaners therefore remove residues in the pores at most but also reduce the active filter area, due to macroscopic cell residues. This cannot be determined by using a porometer, as the porometer only measures the change in flow rate when the pressure is increased and converts this to a pore diameter using the Young–Laplace equation. A reduction in the filter area therefore has no influence on the measurable pore size. In contrast, enzymatic additives break down the macroscopic residues, restoring the active filter area to its original state. At the same time, the abrasive and insoluble particles are deposited in the pores, reducing the pore size and thus lowering the flow rate. The effect of the enlarged active filter area predominates, which is reflected in the reduction in filtration time compared to purely chemical regenerations. Enzymatic additives can therefore sufficiently clean the membrane in dead-end filtration, even with an exposure time of 5 s. Compared to purely mechanical regeneration through backwashing, this can increase the flow rate by 42%, which makes enzymatic additives interesting for regeneration during the filtration of biological particles.

## 4. Conclusions

This paper investigates the regenerative capacity of a 0.8 µm membrane after the filtration of microalgae with x_50.3_ = 10.13 µm and mechanical cake discharge through mechanical, chemical and biological–chemical cleaning. The microalgae suspension with a dry biomass concentration of 1 g L^−1^ is frozen and thawed several times to increase fouling behavior due to AOM and TEP. The aim is to keep the filtration time of 8 L m^−2^ suspension constant after each filtration cycle by means of regeneration. The main findings are summarized in Table 2.

This research shows that enzymatic additives have a positive effect on membrane regeneration compared to the currently available industrial cleaners that are based purely on chemicals. Even in the long term, enzymes are gentler on materials such as PET than pure chemicals. Strong acids and alkalis, in particular, can decompose polymers. Compared to backwashing, enzymes also offer the advantage that no complex mechanical support structure needs to be installed for the fragile polymer membranes. Enzymes are therefore suitable for cleaning membranes, even with a short exposure time. To further increase the flow rate, biological–chemical cleaners without abrasive additives, which can lead to pore blockage, could be used. However, these cleaning agents containing enzymes can only be used in devices without the possibility of remixing the cleaning agent and the solid material as a valuable product. Examples of filter devices include vacuum filters, such as belt filters, drum filters, plane filters and disk filters.

## Figures and Tables

**Table 1:** Mean flow pore size (MFP) of the dry membranes after 10 cycles of filtration and regeneration.

	MFP in µm		MFP in µm
P3-Ultrasil 1% *w*/*w*	0.75 ± 0.03	NaClO 2 mol L^−1^	0.75 ± 0.03
P3-Ultrasil 0.5% *w*/*w*	0.71 ± 0.02	NaClO 1 mol L^−1^	0.77 ± 0.03
P3-Ultrasil 0.1% *w*/*w*	0.66 ± 0.02	NaClO 0.5 mol L^−1^	0.72 ± 0.04
Membrane unused	0.83 ± 0.01		

**Table 2:** A Summary of the main findings for the different regeneration mechanisms.

Regeneration Mechanism	Findings
Mechanical backwashing	With a backwashing pressure of 0.2 bar, the filtration performance can be increased by 350% after 10 cycles. A further increase in the backwashing pressure does increase the filtration performance, but not in proportion to the applied pressure increase.
Cleaning through hydrolysis	Acids (HCl; H_3_NSO_3_) and alkalis (NaOH) with a hydrolytic effect do not have a cleaning effect. This indicates that fouling behavior is not caused by mineral or easily soluble organic residues. The main cause of the increase in filter resistance is therefore the biofilm, which consists of poorly soluble fats and oils.
Cleaning through hydrolysis and surfactants	Both the pure NaClO and the multi-component mixture P3-Ultrasil regenerate the membrane to 5.4 times the throughput compared to that without cleaning. The oxidative effect of NaClO and the dissolving effect of the anionic surfactants in P3-Ultrasil can thus remove the biofilm on the membrane. However, larger cell residues are not removed with these two cleaning agents.
Enzymatic cleaning	Enzymatic additives can increase the flow rate by 61% compared to filtration with the industrial cleaning agent P3-Ultrasil with an identical mass concentration, even at short exposure times of 5 s and at room temperature. Higher temperatures when using enzymes do not result in a further increase in the flow rate. Since the drum filter is a dead-end filtration system and the particles are the valuable product, there is no remixing of the enzymes used with the initial suspension.

## Data Availability

The original contributions presented in this study are included in the article/Supplementary Material. Further inquiries can be directed to the corresponding authors.

## Supplementary Materials

The following supporting information can be downloaded at: https://www.mdpi.com/article/10.3390/membranes16010007/s1. The supplementary materials contain the raw data used to create the figures and diagrams. Furthermore, there are images of the membrane surface from a laser-scanning microscope and a variation in the chemical concentration for HCl and NaOH.

## Abbreviations

The following abbreviations are used in this manuscript:

Symbol	Description	Unit
*d_cap_*	Diameter of the capillary pressure	m
*d*	Diameter	m
*g*	Gravity	m∙s^−2^
*m_B_*	Mass in point B	kg
*P_cap_*	Capillary pressure	Pa
*q_P_*	Line pressure force of the roller	N∙m^−1^
*t*_0_	Filtration time with new membrane	s
*t_cycle_*	Filtration time per cycle	s
*t**	Normalized filtration time	-
x_10.3_	Mass/volume-related diameter	µm
x_50.3_	Mass/volume-related modal value	µm
x_90.3_	Mass/volume-related diameter	µm
*γ_L_*	Surface tension of the liquid	N∙m^−1^
Δp	Pressure difference	Pa
*η_L_*	Dynamic viscosity of the liquid	Pa∙s
*θ*	Contact angle of the liquid	°
AOM	Algal organic matter	
HCl	Hydrochloric acid	
H_3_NSO_3_	Sulfamic acid	
MFP	Mean flow pore size	
NaClO	Sodium hypochlorite	
NaOH	Sodium hydroxide	
PET	Polyethylene terephthalate	
TEP	Transparent exopolymer particles	

## References

[1] Borowitzka M.A., Beardall J., Raven J.A. (2016). The Physiology of Microalgae.

[2] Chapman R.L. (2013). Algae: The World’s Most Important “Plants”—An Introduction. Mitig. Adapt. Strateg. Glob. Change.

[3] Tua C., Ficara E., Mezzanotte V., Rigamonti L. (2021). Integration of a Side-Stream Microalgae Process into a Municipal Wastewater Treatment Plant: A Life Cycle Analysis. J. Environ. Manag..

[4] Senatore V., Buonerba A., Zarra T., Oliva G., Belgiorno V., Boguniewicz-Zablocka J., Naddeo V. (2021). Innovative Membrane Photobioreactor for Sustainable CO2 Capture and Utilization. Chemosphere.

[5] Norton T.A., Melkonian M., Andersen R.A. (1996). Algal Biodiversity. Phycologia.

[6] Kim S.-K., Ravichandran Y.D., Khan S.B., Kim Y.T. (2008). Prospective of the Cosmeceuticals Derived from Marine Organisms. Biotechnol. Bioprocess Eng..

[7] Spolaore P., Joannis-Cassan C., Duran E., Isambert A. (2006). Commercial Applications of Microalgae. J. Biosci. Bioeng..

[8] Rosenberg J.N., Oyler G.A., Wilkinson L., Betenbaugh M.J. (2008). A Green Light for Engineered Algae: Redirecting Metabolism to Fuel a Biotechnology Revolution. Curr. Opin. Biotechnol..

[9] Leite G.B., Abdelaziz A.E.M., Hallenbeck P.C. (2013). Algal Biofuels: Challenges and Opportunities. Bioresour. Technol..

[10] Novoa A.F., Vrouwenvelder J.S., Fortunato L. (2021). Membrane Fouling in Algal Separation Processes: A Review of Influencing Factors and Mechanisms. Front. Chem. Eng..

[11] Khanzada N.K., Al-Juboori R.A., Khatri M., Ahmed F.E., Ibrahim Y., Hilal N. (2024). Sustainability in Membrane Technology: Membrane Recycling and Fabrication Using Recycled Waste. Membranes.

[12] Rodríguez-Sáez L., Landaburu-Aguirre J., García-Calvo E., Molina S. (2024). Application of Recycled Ultrafiltration Membranes in an Aerobic Membrane Bioreactor (aMBR): A Validation Study. Membranes.

[13] Schaefer A., Andritsos N., Karabelas A., Hoek E., Schneider R., Nyström M. (2004). Fouling in Nanofiltration. Nanofiltration—Principles and Applications.

[14] Anlauf H. (2019). Wet Cake Filtration: Fundamentals, Equipment, and Strategies.

[15] Rajendran D.S., Devi E.G., Subikshaa V.S., Sethi P., Patil A., Chakraborty A., Venkataraman S., Kumar V.V. (2025). Recent Advances in Various Cleaning Strategies to Control Membrane Fouling: A Comprehensive Review. Clean Technol. Environ. Policy.

[16] Marudhupandi T., Sathishkumar R., Kumar T.T.A. (2016). Heterotrophic Cultivation of Nannochloropsis Salina for Enhancing Biomass and Lipid Production. Biotechnol. Rep..

[17] Najjar Y.S.H., Abu-Shamleh A. (2020). Harvesting of Microalgae by Centrifugation for Biodiesel Production: A Review. Algal Res..

[18] Zhang Y., Zhao Y., Chu H., Zhou X., Dong B. (2014). Dewatering of Chlorella Pyrenoidosa Using Diatomite Dynamic Membrane: Filtration Performance, Membrane Fouling and Cake Behavior. Colloids Surf. B Biointerfaces.

[19] Chen X., Huang C., Liu T. (2012). Harvesting of Microalgae *Scenedesmus* sp. Using Polyvinylidene Fluoride Microfiltration Membrane. Desalination Water Treat..

[20] Liao Y., Bokhary A., Maleki E., Liao B. (2018). A Review of Membrane Fouling and Its Control in Algal-Related Membrane Processes. Bioresour. Technol..

[21] Zhang Y., Wang X., Jia H., Fu B., Xu R., Fu Q. (2019). Algal Fouling and Extracellular Organic Matter Removal in Powdered Activated Carbon-Submerged Hollow Fiber Ultrafiltration Membrane Systems. Sci. Total Environ..

[22] Zhang Y., Fu Q. (2018). Algal Fouling of Microfiltration and Ultrafiltration Membranes and Control Strategies: A Review. Sep. Purif. Technol..

[23] Ehrfeld E., Bott R. (1990). Continuous Filtration without Gas Throughput—Using Membrane Filter Media. Filtr. Sep..

[24] Shen Y., Badireddy A.R. (2021). A Critical Review on Electric Field-Assisted Membrane Processes: Implications for Fouling Control, Water Recovery, and Future Prospects. Membranes.

[25] Liang Y.Y. (2025). Role of Spacers in Osmotic Membrane Desalination: Advances, Challenges, Practical and Artificial Intelligence-Driven Solutions. Process Saf. Environ. Prot..

[26] Ibrahim A.A., Dalle M.-A., Janasz F., Leyer S. (2024). Novel Spacer Geometries for Membrane Distillation Mixing Enhancement. Desalination.

[27] Chen J.P., Kim S.L., Ting Y.P. (2003). Optimization of Membrane Physical and Chemical Cleaning by a Statistically Designed Approach. J. Membr. Sci..

[28] Zhou Z., Meng F., Lu H., Li Y., Jia X., He X. (2014). Simultaneous Alkali Supplementation and Fouling Mitigation in Membrane Bioreactors by On-Line NaOH Backwashing. J. Membr. Sci..

[29] Maskooki A., Mortazavi S.A., Maskooki A. (2010). Cleaning of Spiralwound Ultrafiltration Membranes Using Ultrasound and Alkaline Solution of EDTA. Desalination.

[30] Bhatia S. (2018). Introduction to Pharmaceutical Biotechnology. Volume 2: Enzymes, Proteins and Bioinformatics. IOP Expanding Physics.

[31] Muñoz-Aguado M.J., Wiley D.E., Fane A.G. (1996). Enzymatic and Detergent Cleaning of a Polysulfone Ultrafiltration Membrane Fouled with BSA and Whey. J. Membr. Sci..

[32] Chen D., Columbia M. (2011). Enzymatic Control of Alginate Fouling of Dead-End MF and UF Ceramic Membranes. J. Membr. Sci..

[33] Shirazi S., Lin C.-J., Chen D. (2010). Inorganic Fouling of Pressure-Driven Membrane Processes—A Critical Review. Desalination.

[34] Lorenz R.T. (1999). A Technical Review of *Haematococcus* Algae. NatuRose Tech. Bull..

[35] Do Carmo Cesário C., Soares J., Cossolin J.F.S., Almeida A.V.M., Bermudez Sierra J.J., De Oliveira Leite M., Nunes M.C., Serrão J.E., Martins M.A., Dos Reis Coimbra J.S. (2022). Biochemical and Morphological Characterization of Freshwater Microalga *Tetradesmus obliquus* (Chlorophyta: Chlorophyceae). Protoplasma.

[36] Melin T., Rautenbach R. (2007). Membranverfahren: Grundlagen der Modul- und Anlagenauslegung.

[37] Ding J., Wang S., Xie P., Zou Y., Wan Y., Chen Y., Wiesner M.R. (2020). Chemical Cleaning of Algae-Fouled Ultrafiltration (UF) Membrane by Sodium Hypochlorite (NaClO): Characterization of Membrane and Formation of Halogenated by-Products. J. Membr. Sci..

[38] Achaw O.-W., Danso-Boateng E. (2021). Soaps and Detergents. Chemical and Process Industries.

[39] Bächle V., Gleiß M., Nirschl H. (2024). Influence of Particles on the Roller Discharge of Thin-Film Filtration without Gas Throughput. Chem. Eng. Technol..

[40] Lam Z., Anlauf H., Nirschl H. (2021). Thin-Film Filtration of Difficult-to-Filter Suspensions Using Polymeric Membranes. Chem. Eng. Technol..

[41] Zhou H., Ji C., Li J.-Q., Hu Y.-X., Xu X.-H., An Y., Cheng L.-H. (2021). Understanding the Interaction Mechanism of Algal Cells and Soluble Algal Products Foulants in Forward Osmosis Dewatering. J. Membr. Sci..

[42] Psoch C., Schiewer S. (2006). Anti-Fouling Application of Air Sparging and Backflushing for MBR. J. Membr. Sci..

[43] Jander G., Jahr K.F., Schulze G., Simon J. (2003). Maßanalyse: Theorie und Praxis der Titrationen mit Chemischen und Physikalischen Indikationen.

[44] Ding J., Wan Y., Zou Y., Wang S., Huang X., Xie P. (2023). Removal of Membrane Fouling and Control of Halogenated By-Products by a Combined Cleaning Process with Peroxides and Sodium Hypochlorite. Water.

[45] Rudolph G., Schagerlöf H., Morkeberg Krogh K.B., Jönsson A.-S., Lipnizki F. (2018). Investigations of Alkaline and Enzymatic Membrane Cleaning of Ultrafiltration Membranes Fouled by Thermomechanical Pulping Process Water. Membranes.

[46] Ciftci D., Flores R.A., Saldaña M.D.A. (2018). Cellulose Fiber Isolation and Characterization from Sweet Blue Lupin Hull and Canola Straw. J. Polym. Environ..

