# Supplementary material for: Characterization of the Regenerative Capacity of Membranes in the Presence of Fouling by Microalgae Using Detergents
Source: Membranes (Basel). 2025 Dec 26;16(1):7. doi: 10.3390/membranes16010007 (PMC12844485; doi:10.3390/membranes16010007)
Supplement: Supplementary file 1 [file membranes-16-00007-s001.zip › membranes-4061529-supplementary.pdf]

Supplementary Materials

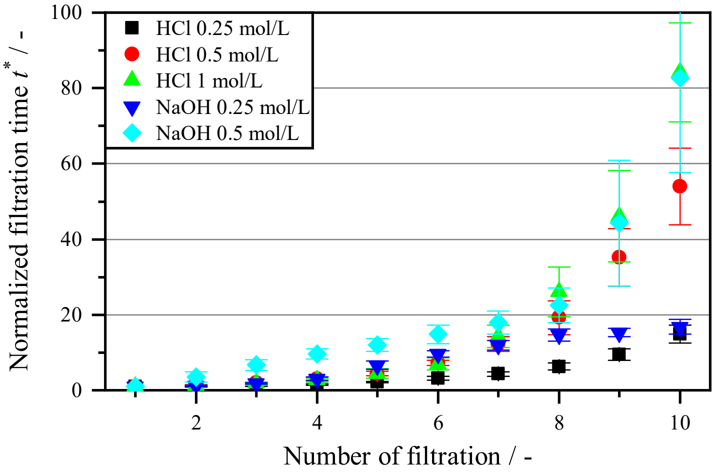

Figure S1. Increase in filtration time with regeneration using HCl and NaOH in different concentrations and an exposure time of 5 seconds after each cycle.

Additional images of the membrane surface using a laser scanning microscope and different cleaning conditions after 10 cycles. Nevertheless, microscope images only show small areas, which is why surface contamination can vary across the entire membrane. This is also related to the roller discharge for cake removal. Therefore, an overall view of the flow/filtration time is more representative.

(a) New membrane

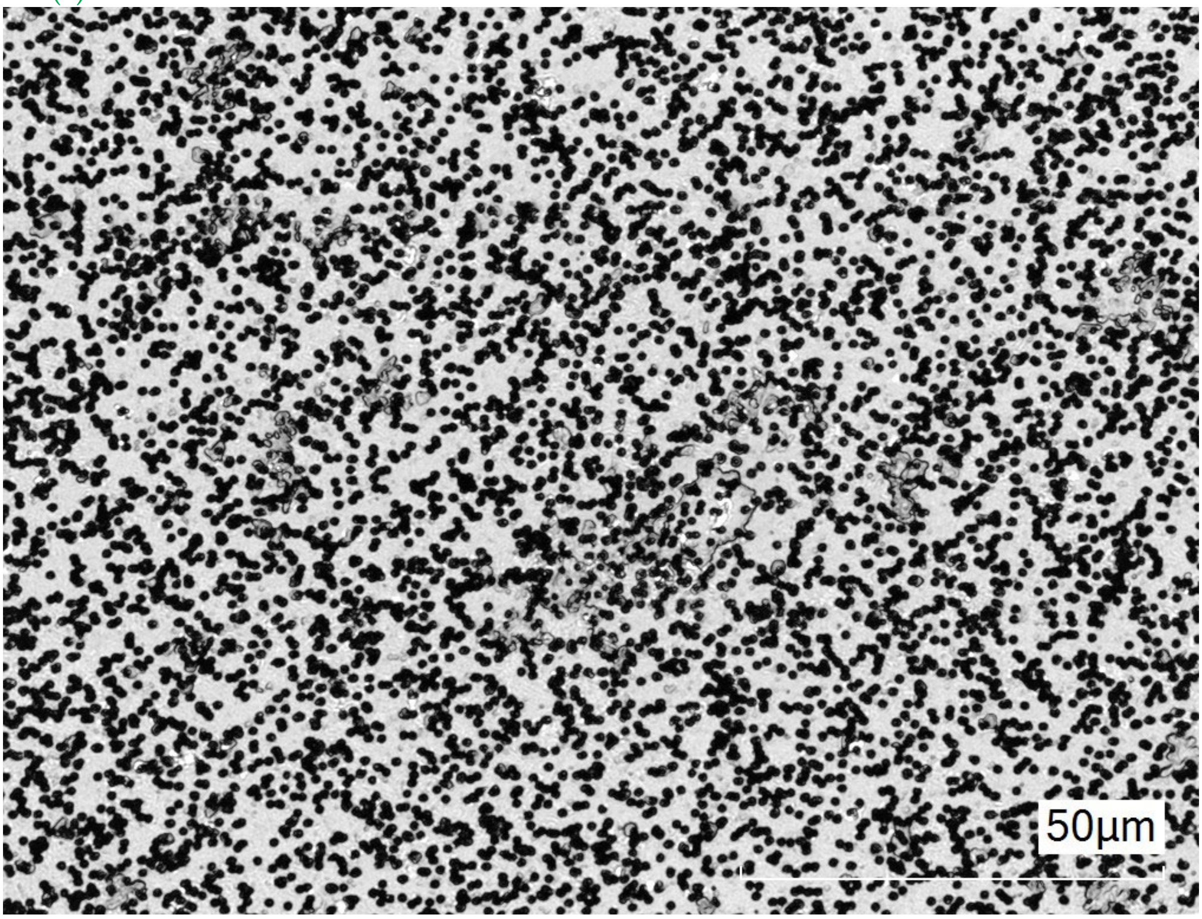

(b) HCl 0.5 mol L^{-1}

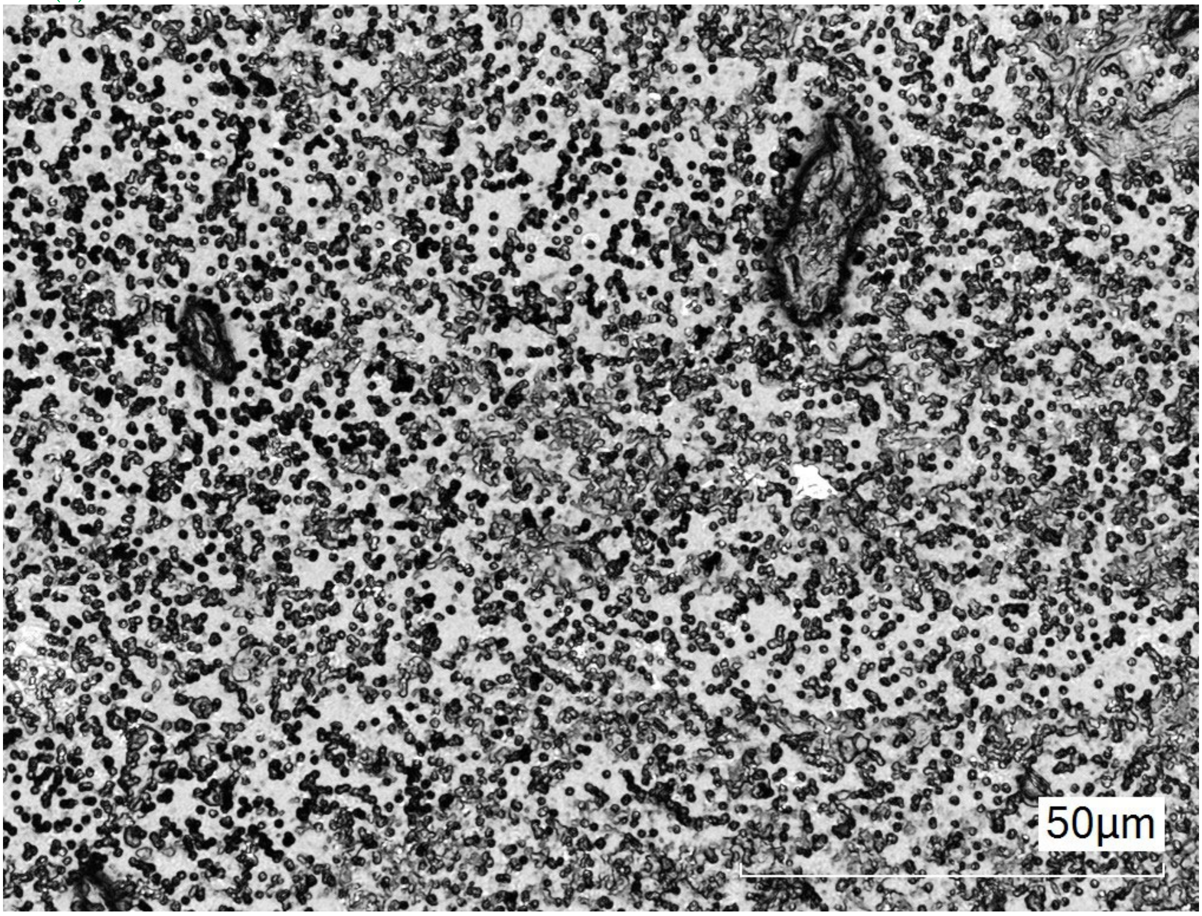

(c) H_3NSO_3 0.5 mol L^{-1}

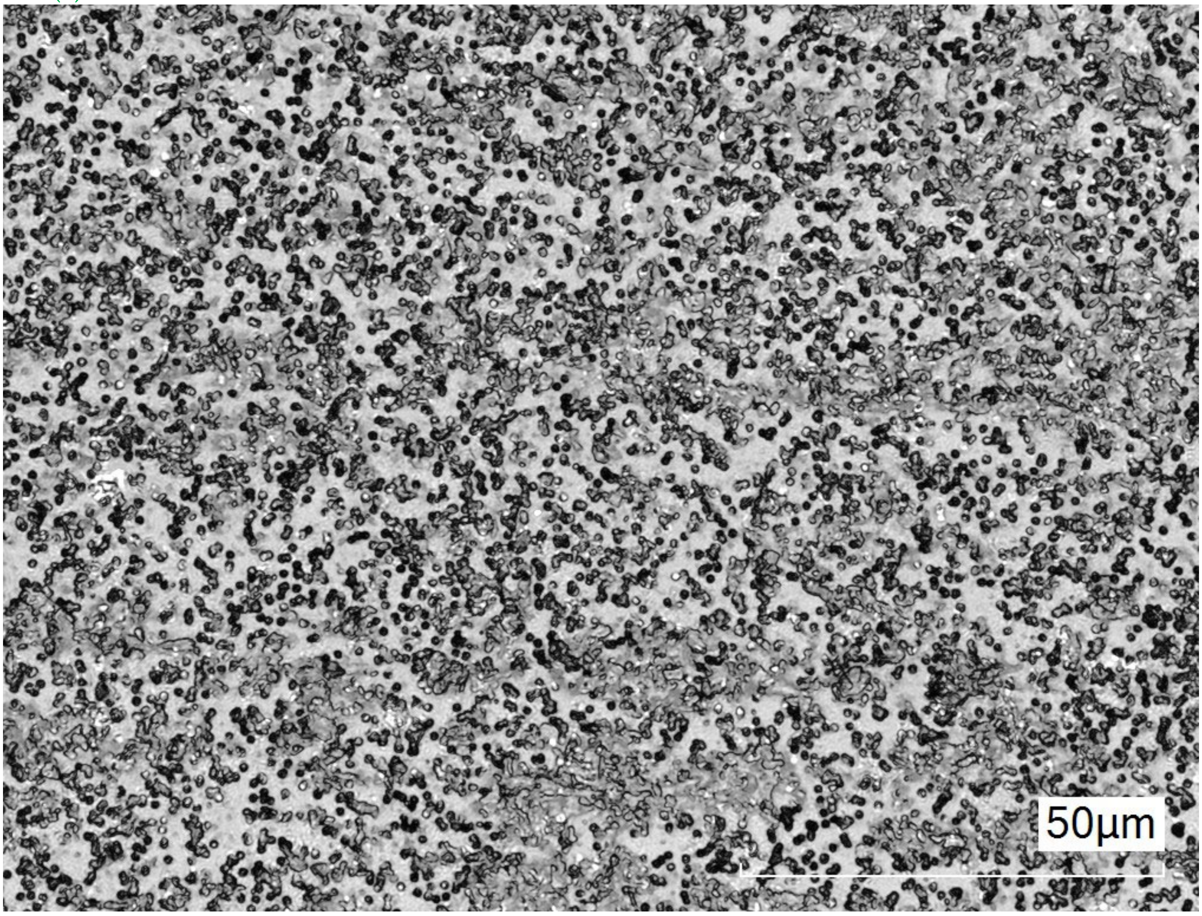

(d) NaClO 0.5 mol L^{-1}

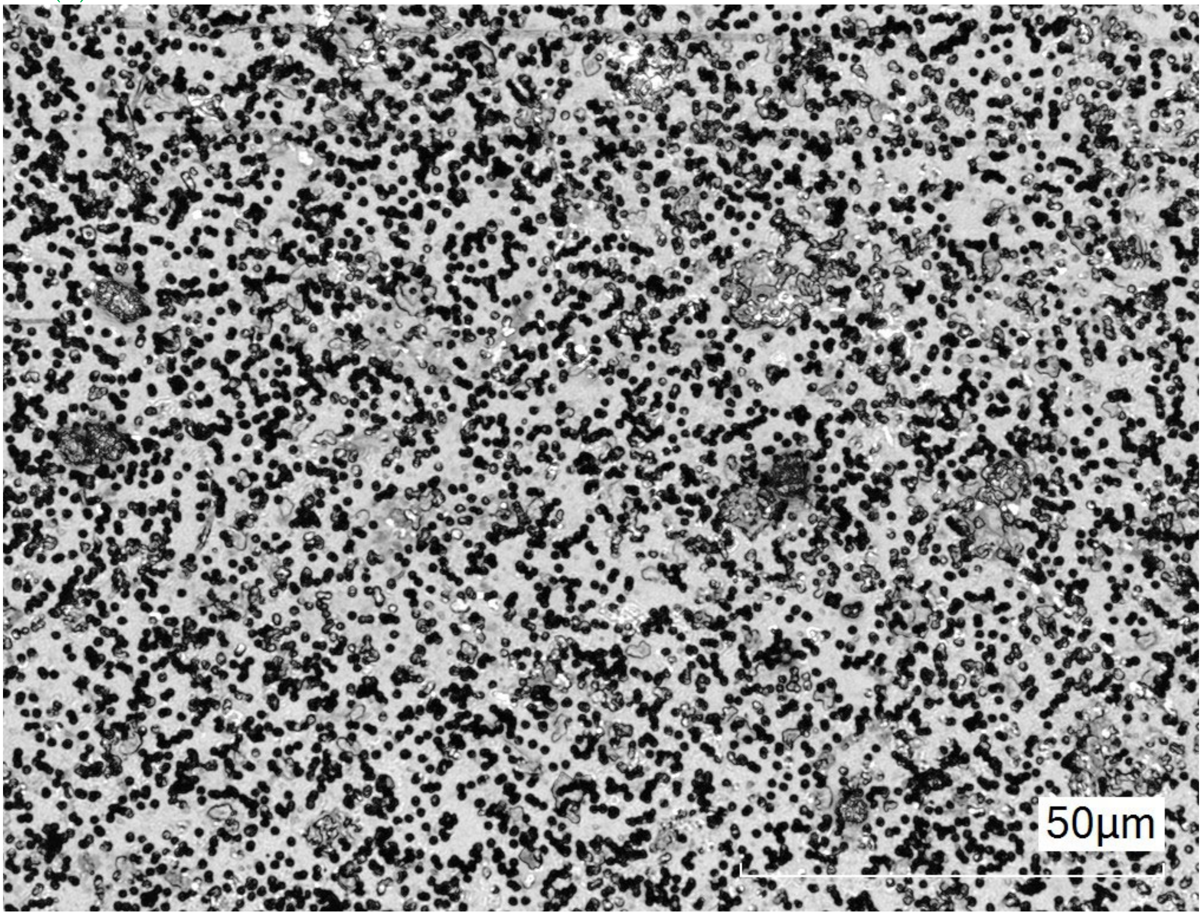

(e) P3-Ultrasil 0.5 % w/w

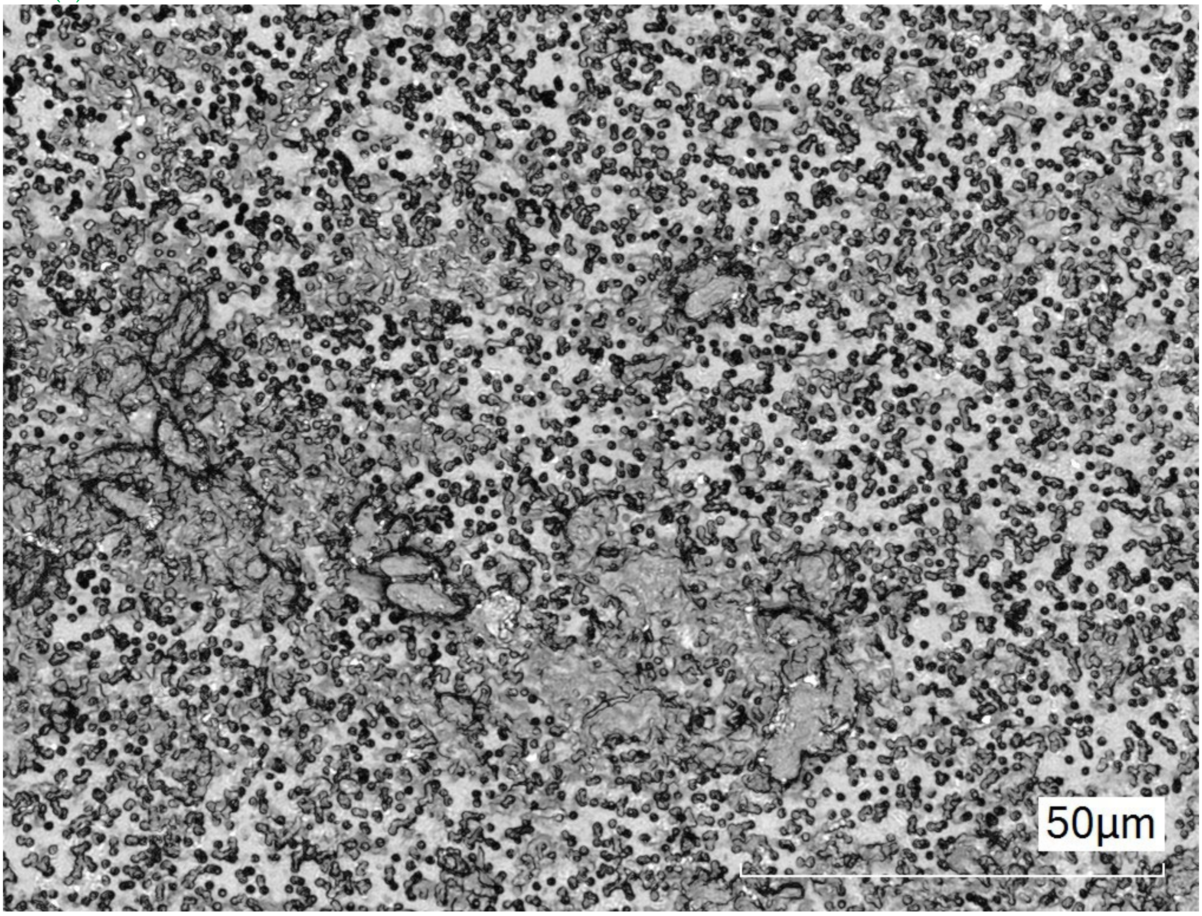

(f) Laundry detergent 40°C

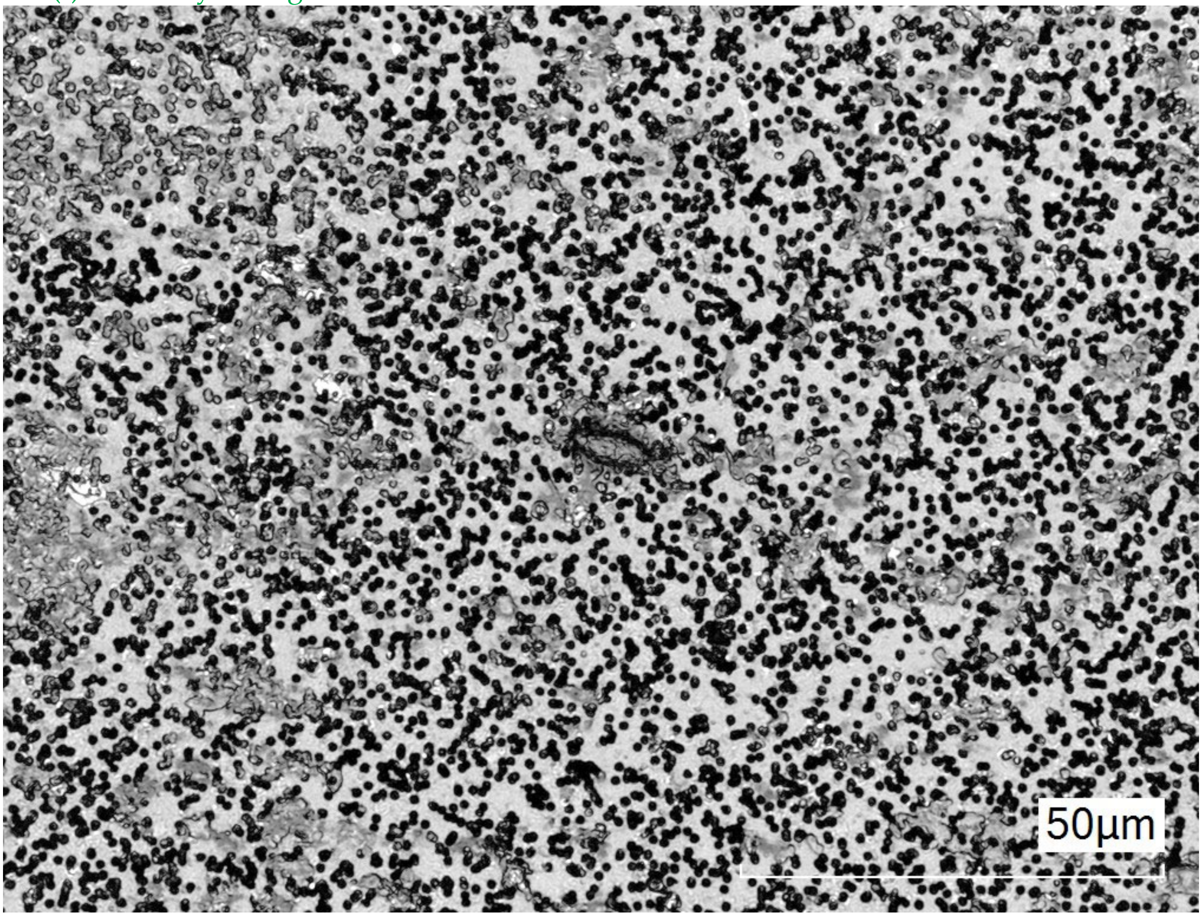

(g) Dishwasher detergent 20°C

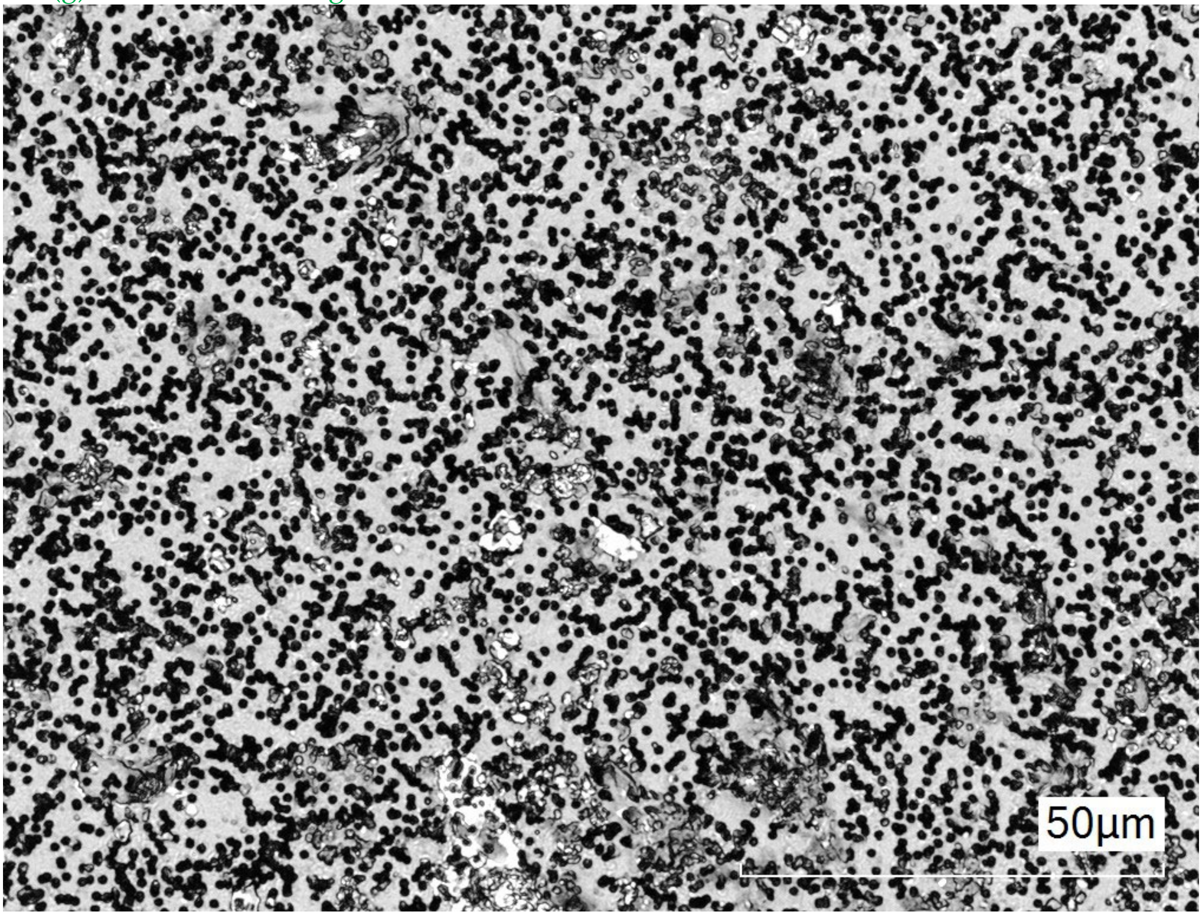

Figure S2. Microscope images of the membrane surface after 10 cycles and different detergents.
